# EVIDENCE THAT CONSERVED ESSENTIAL GENES ARE ENRICHED FOR PRO-LONGEVITY FACTORS

**DOI:** 10.1093/geroni/igad104.2487

**Published:** 2023-12-21

**Authors:** Alaattin Kaya

**Affiliations:** Virginia Commonwealth University, Richmond, Virginia, United States

## Abstract

At the cellular level, many aspects of aging are conserved across species. This has been demonstrated by numerous studies in simple model organisms like Saccharomyces cerevisiae, Caenorhabdits elegans, and Drosophila melanogaster. Functional genomic studies in these models have contributed enormously to our understanding of conserved genetic pathways influencing aging in evolutionarily divergent organisms. From this perspective, essential genes that are evolutionarily constrained and functionally conserved should be more relevant to the regulation and evolution of aging. However, because most genetic screens examine loss of function mutations or decreased expression of genes through reverse genetics, essential genes have often been overlooked as potential modulators of the aging process. By taking the approach of increasing the expression level of conserved essential genes, we found that 23% of these genes resulted in increased replicative lifespan in S. cerevisiae. This is greater than the ~3.5% of genes found to affect lifespan upon deletion, suggesting that activation of essential genes may have a relatively disproportionate effect on increasing lifespan. These essential prolongevity genes function in a variety of pathways and complexes highlighting both known and previously unknown longevity pathways and their previously unknown essential components. The results of our experiments demonstrate that essential gene overexpression is a rich, relatively unexplored means of increasing eukaryotic lifespan.

